# Long-term oncological outcomes of low anterior resection for rectal cancer with and without preservation of the left colic artery: a retrospective cohort study

**DOI:** 10.1186/s12885-021-07848-y

**Published:** 2021-02-17

**Authors:** Yuwen Luo, Rongjiang Li, Deqing Wu, Jun Zeng, Junjiang Wang, Xianzhe Chen, Chengzhi Huang, Yong Li, Xueqing Yao

**Affiliations:** 1 The Second School of Clinical Medicine, Southern Medical University, Guangzhou, 510082 People’s Republic of China; 2 Department of General Surgery, Guangdong Provincial People’s Hospital, Guangdong Academy of Medical Sciences, Guangzhou, 510080 People’s Republic of China; 3 Department of General Surgery, Baoan Central Hospital, The Fifth Affiliated hospital of Shenzhen University, Shenzhen, 518000 People’s Republic of China; 4 School of Medicine, South China University of Technology, Guangzhou, Guangdong 510006 People’s Republic of China; 5 School of Biology and Biological Engineering, South China University of Technology, Guangzhou, Guangdong 510006 People’s Republic of China

**Keywords:** Rectal cancer, Left colic artery, Inferior mesenteric artery, Long-term oncologic outcomes

## Abstract

**Background:**

There is uncertainty in the literature about preserving the left colic artery (LCA) during low anterior resection for rectal cancer. We analyzed the effect of preserving the LCA on long-term oncological outcomes.

**Methods:**

We retrospectively collected clinicopathological and follow-up details of patients who underwent low anterior resection for rectal cancer in the General Surgery Department of Guangdong Provincial People’s Hospital, from January 2014 to December 2015. Cases were divided into low ligation (LL), LCA preserved, or high ligation (HL), LCA not preserved, of the inferior mesenteric artery. The 5-year overall survival (OS) and disease-free survival (DFS) rates were compared between the two groups.

**Results:**

Altogether, there were 221 and 295 cases in the LL group and HL groups, respectively. Operating time in the LL group was significantly longer than in the HL group (224.7 vs. 211.7 min, *p* = 0.039). Postoperative 30-day mortality, early complications including anastomotic leakage showed no significant differences between the LL and HL groups (postoperative 30-day mortality, 0.9% LL, 1.4% HL, *p* = 0.884; early complications, 41.2% LL, 38.3% HL, *p* = 0.509; anastomotic leakage 8.6% LL, 13.2% HL, *p* = 0.100). The median follow-up periods were 51.4 (7–61) months in the LL group and 51.2 (8–61) months in the HL group. During follow-up, the percentages of patients who died, had local recurrence, or had metastases were 39.8, 7.7, and 38.5%, respectively, in the LL group and 39, 8.5, and 40%, respectively, in the HL group; these differences were not significant (all *p* > 0.05). The 5-year OS and DFS were 69.6 and 59.6% in the LL group, respectively, and 69.1 and 56.2% in the HL group, respectively; these differences were not significant (all *p* > 0.05). After stratification by tumor-node-metastasis stage, the difference between the 5-year OS and DFS for stages I, II, and III cancer were not significant (all *p* > 0.05).

**Conclusions:**

The long-term oncological outcomes of LL group are comparable with HL group. LL cannot be supported due to the absence of lower complication rates and the longer operating times.

## Background

Colorectal cancer is the third most diagnosed cancer in men and the second in women [1]. In recent years, colorectal cancer management includes surgery supplemented by radiotherapy and chemotherapy [2, 3]. Anatomically, the colon and rectum have distinct locations, blood supply, drainage and innervation. These differences result in dissimilarities in the invasive growth of the primary tumor as well as surgical approaches and treatment outcomes [4]. Rectal cancer surgery is methodically more complex, and the surgical demands increase with the depth of the aboral tumor position and proximity to the sphincter muscle [5, 6]. During low anterior resection for rectal cancer, the inferior mesenteric artery (IMA) can be treated in two ways: one is to identify and preserve the left colic artery (LCA) while ligating the IMA after bifurcation of the LCA, and the other is to directly ligate the root of the IMA without preserving the LCA [7]. According to Bertrand’s study [8, 9], the branches of the IMA are subject to wide interindividual variation making it difficult, if not impossible, to replicate surgery based on anatomy. It is thought that preserving the LCA maintains a better blood supply [10], which leads to a lower anastomotic leakage rate [11]. However, this technique may lead to greater anastomotic tension, prolonged operation time, and more technical challenges [6].

Many studies, including meta-analyses, suggest that preserving the LCA may reduce the incidence of anastomose-related complications but has no effect on the long-term oncologic outcomes [10–13]. A randomized, multicenter, controlled trial showed that low ligation (LL) of the IMA in laparoscopic rectal cancer surgery resulted in better genitourinary function preservation without affecting initial oncological outcomes, while high ligation (HL) of the IMA did not increase the anastomotic leak rate [14]. However, laparotomy cases were excluded, and the effects of high or low ligation of the IMA on long-term oncologic outcomes were not reported. In Fujii’s study [15], laparotomy cases were included, and the results showed that the IMA ligation level was unrelated to anastomotic leakage. Further, there was no significant difference observed in the long-term outcomes in patients with or without LCA preservation. Since the benefits of LCA preservation are still debatable, we analyzed the data obtained from patients who underwent low anterior resection for rectal cancer in the General Surgery Department of Guangdong Provincial People’s Hospital to evaluate whether preservation of the LCA affected the early complication rate and long-term oncologic outcomes.

## Methods

### Patients

Inclusion criteria: (1) 18 years of age and over; (2) low anterior resection for rectal cancer; (3) postoperative pathological diagnosis of rectal adenocarcinoma; and (4) informed consent signed prior to surgery. Exclusion criteria: (1) recurrent rectal cancer; (2) emergency surgery; (3) preoperative and intraoperative detection of distant organ metastases or extensive implantation metastases in the abdominal cavity; (4) palliative surgery; (5) a postoperative pathology report that showed residual cancer cells at the proximal or distal resection margin; (6) no standard chemotherapy for tumor-node-metastasis (TNM) staging II or III after surgery; (7) synchronous colorectal carcinoma and other organ tumors; and (8) incomplete case data.

Based on the above criteria, we retrospectively collected data from the medical records of patients who had a low anterior resection for rectal cancer at Guangdong Provincial People’s Hospital, from January 2014 to December 2015. A total of 516 from 635 cases were included in this study; in 221 cases, the LCA was preserved intraoperatively, while in the remaining 295 cases, the LCA was not preserved.

### Surgical procedure

All patients underwent total mesorectal excision, or partial mesorectal excision, plus D3 lymph node dissection with sphincter preservation [16]. For the HL group, the IMA was ligated and divided 2 cm from its origin. The inferior mesenteric vein was ligated and divided below the pancreatic margin. For the LL group, the LCA was identified and preserved while low ligation of the IMA (the superior hemorrhoidal artery) was performed. Lymphadenectomy was performed medially along the IMA until reaching 2 cm from the aorta. After resection of the tumor, the bowel proximal to the pubic symphysis was checked to ensure it was free of tension. Splenic flexure mobilization was performed if the bowel was not free. To reconstruct the gastrointestinal tract, an end-to-end colorectal anastomosis was made. If the surgeon thought it was necessary, based on a technical evaluation of the quality of the anastomosis, a diverting ileostomy was added.

### Postoperative adjuvant chemotherapy

Patients with stage II or III adenocarcinoma were treated with the XELOX regimen (oxaliplatin, capecitabine) of chemotherapy for six to eight courses after surgery.

### Surgical parameters and postoperative follow-up

Surgical parameters recorded were: operation time, presence of splenic flexure mobilization, intraoperative complications, 30-day postoperative mortality, 30-day postoperative complications (classified as mild or severe; Clavien-Dindo classification ≤ II was considered a mild complication, ≥ III was considered a severe complication), anastomotic level from the anal verge, anastomotic leak classification (according to the standard of The International Study Group of Rectal Cancer, ISREC. In simple terms, Grade A anastomotic leakage results in no change in patients’ management, whereas grade B leakage requires active therapeutic intervention but is manageable without re-laparotomy and Grade C anastomotic leakage requires re-laparotomy [17]), length of distal margin, number of lymph nodes harvested, and the number of positive lymph nodes.

Periodic patient follow-up with office visits for 5 years: every 3–6 months for 2 years after surgery; every 6–12 months for 3 to 5 years after surgery; once a year for 5 years after surgery. The follow-up visit included a physical examination, carcinoembryonic antigen measurement, a computed tomography scan, and colonoscopy. Confirmation of recurrence required imaging or pathological evaluation.

### Statistical analyses

Statistical analyses were performed using SPSS (Statistical Product and Service Solutions version 22.0 for Windows, Armonk, NY, USA: IBM Corp). Quantitative data were described using the mean ± standard deviation, and t-tests or rank sum tests were used to test the hypothesis. Categorical data were described by the number of cases and percentages, and Chi-square (χ2) or Fisher’s exact tests were used to test the hypothesis. The Kaplan-Meier method was used to estimate survival, and the log-rank test was used to evaluate differences between the survival curves. Statistical significance was considered to exist when *p* < 0.05.

## Results

### Patient clinical characteristics

A total of 516 rectal cancer patients, including 346 (67.1%) men and 170 (32.9%) women, were enrolled in this study. Of these cases, 221 (42.8%) were in the LL group and 295 (57.2%) were in the HL group. The clinicopathological characteristics of the two groups are provided in Table 1. There were no statistically significant differences between the two groups with regards to sex, age, body mass index (BMI), tumor location, neoadjuvant therapy, or postoperative adjuvant treatment.

**Table 1:** Patient Clinical Characteristics at Baseline

	HL (*n* = 295)	LL (*n* = 221)	*P* value
Sex [n (%)]			0.732
Male	196 (66.4)	150 (67.9)	
Female	99 (33.6)	71 (32.1)	
Age: years	63.3 ± 10.7	64.4 ± 9.8	0.471
BMI: kg/m^2^	22.3 ± 3.0	22.4 ± 3.1	0.832
ASA classification			0.117
I	151 (51.2)	97 (43.9)	
II	101 (34.2)	78 (35.3)	
III	43 (14.6)	46 (20.8)	
Preoperative Hb: g/L	129.7 ± 11.4	131.6 ± 10.7	0.285
Preoperative Alb: g/L	34.8 ± 3.4	35.0 ± 3.5	0.657
Tumor location [n (%)]			0.459
Upper rectum ^a^	77 (26.1)	66 (29.9)	
Middle rectum ^b^	133 (45.1)	101 (45.7)	
Lower rectum ^c^	85 (28.8)	54 (24.4)	
Neoadjuvant therapy ^d^ [n (%)]	65 (22.0)	46 (20.8)	0.739
Postoperative adjuvant treatment [n (%)]	89 (30.2)	71 (32.1)	0.634

*Abbreviations*: *BMI* Body mass index, *Hb* Hemoglobin, *Alb* Albumin, *HL* High ligation, *LL* Low ligation, *ASA classification* American Society of Anesthesiologists Classification

^a^ > 10 cm

^b^10–5 cm

^c^ < 5 cm

^d^ Neoadjuvant chemotherapy or neoadjuvant chemoradiotherapy

### Surgical outcomes

The surgical outcomes and early complications are presented in Table 2. The operating time in the LL group was significantly longer than that of the HL group (224.7 min vs. 211.7 min, respectively; *p* = 0.039). There was no significant difference in operative bleeding between the LL and HL groups (76 ml vs. 70 ml, respectively; *p* = 0.252). Splenic flexure mobilization in the LL group was significantly higher than that in the HL group (13.1% vs. 6.1%, respectively; *p* = 0.006). The rate of protective ileostomies in the LL group was 29.9 and 24.1% in the HL group (*p* = 0.140), which showing no significant difference. The anastomotic level from the anal verge in the LL group was 5.9 ± 2.0 cm, and in the HL group was 5.6 ± 2.1 cm (*p* = 0.418). The 30-day postoperative mortality was 0.9% in the LL group and 1.4% in the HL group (*p* = 0.884). In the LL group, 29.0 and 12.2% experienced mild and severe complications, respectively, while in the HL group, 26.4 and 11.9% experienced mild and severe complications, respectively (*p* = 0.788). Anastomotic leakage occurred in 2.3 and 1.8% of ISREC grade B and C cases, respectively, in the LL group and 2.4 and 2.4% of ISREC grade B and C cases, respectively, in the HL group (*p* = 0.202). Reoperation occurred in 1.8% of the LL group and 2.4% of the HL group (*p* = 0.691).

**Table 2:** Surgical outcomes and early complications

	HL (*n* = 295)	LL (*n* = 221)	*P* value
Surgical approach [n (%)]			0.425
Laparoscopic surgery	250 (84.7)	192 (86.9)	
Open surgery	40 (13.6)	23 (10.4)	
Laparoscopic conversed to open surgery	5 (1.7)	6 (2.7)	
Surgical resection			0.454
PME	89 (30.2)	60 (27.1)	
TME	206 (69.8)	161 (72.9)	
Operating time: minutes	211.7 ± 44.5	224.7 ± 32.7	0.039
Operative bleeding: ml	70.0 ± 23.5	76.0 ± 39.4	0.252
Splenic flexure mobilization [n (%)]	18 (6.1)	29 (13.1)	0.006
Postoperative mortality [n (%)]	4 (1.4)	2 (0.9)	0.884 ^a^
Postoperative complication morbidity ^b^ [n (%)]			0.788
Mild ^c^	78 (26.4)	64 (29.0)	
Severe ^d^	35 (11.9)	27 (12.2)	
Anastomotic level from anal verge: cm	5.6 ± 2.1	5.9 ± 2.0	0.418
Protective ileostomies [n (%)]	71 (24.1)	66 (29.9)	0.140
Anastomotic bleeding [n (%)]	16 (5.4)	13 (5.9)	0.823
Anastomotic stenosis [n (%)]	7 (2.4)	5 (2.3)	0.934
Anastomotic leakage ^e^ [n (%)]			0.202
Grade B	32 (10.8)	14 (6.3)	
Grade C	7 (2.4)	5 (2.3)	
Reoperation	7 (2.4)	4 (1.8)	0.691 ^a^
Postoperative hospital stay: days	6.4 ± 2.4	7.1 ± 4.1	0.155

*Abbreviations*: *HL* High ligation, *LL* Low ligation, *PME* Partial mesorectal excision, *TME* Total mesorectal excision

^a^ Using Fisher’s Exact Probability Tests

^b^ Within 30 days of discharge

^c^ Clavien-Dindo classification ≤ II was considered as mild complication,

^d^ Clavien-Dindo classification≥ III was considered as Severe complication

^e^ According to International Study Group of Rectal Cancer, ISREC

### Pathology outcomes

The pathology outcomes are presented in Table 3. The length of the distal margin in the LL group was 2.6 ± 0.7 cm and was 2.7 ± 0.8 cm in the HL group (*p* = 0.429). The number of lymph nodes harvested in the LL group was 18.8 ± 9.6 and was 17.0 ± 6.6 in the HL group (*p* = 0.111). The number of positive lymph nodes and tumors as per TNM staging was not significantly different between the two groups (all *p* > 0.05).

**Table 3:** Pathology Outcomes

	HL (*n* = 295)	LL (*n* = 221)	*P* value
Distal margin: cm	2.7 ± 0.8	2.6 ± 0.7	0.429
Number of lymph nodes harvested	17.0 ± 6.6	18.8 ± 9.6	0.111
Number of stage III positive lymph nodes	4.0 ± 1.9	3.9 ± 1.9	0.810
Tumor TNM ^a^ staging			0.807
I	39 (13.2)	26 (11.8)	
II	126 (42.7)	92 (41.6)	
III	130 (44.1)	103 (46.6)	

*Abbreviations*: *HL* High ligation, *LL* Low ligation

^a^According to the Cancer Staging Manual, 7th edition

### Follow-up outcomes

The Long-term outcomes are presented in Table 4. Altogether, 91.5% patients completed follow-up in the LL group, median follow-up time was 51.4 (7–61) months, and 89.2% patients completed follow-up in the HL group, median follow-up time was 51.2 (8–61) months. During follow-up, the numbers of patients who died, had local recurrence, or had metastases were 88 (39.8%), 17 (7.7%), and 85 (38.5%), respectively, in the LL group and 115 (39.0%), 25 (8.5%), and 118 (40.0%), respectively, in the HL group. The differences were not significant (all *p* > 0.05).

**Table 4:** Long-term results

	HL (*n* = 295)	LL (*n* = 221)	*P* value
Follow-up time: month (min–max)	51.2 (8–61)	51.4 (7–61)	0.887
5-year overall survival rate			
All stages	69.1%	69.6%	0.942
Stage I	91.3%	85.7%	0.633
Stage II	75.9%	75.0%	0.955
Stage III	56.5%	61.4%	0.657
5-year disease-free survival rate			
All stages	56.2%	59.6%	0.570
Stage I	77.4%	80.0%	0.796
Stage II	62.1%	66.7%	0.512
Stage III	44.6%	49.3%	0.753
Loss of follow-up [n (%)]	32 (10.8)	21 (9.5)	0.618
Local recurrence [n (%)]	25 (8.5)	17 (7.7)	0.748
Tumor metastasis [n (%)]	118 (40.0)	85 (38.5)	0.723
Death [n (%)]	115 (39.0)	88 (39.8)	0.848

*Abbreviations*: *HL* High ligation, *LL* Low ligation

**Fig. 1:**
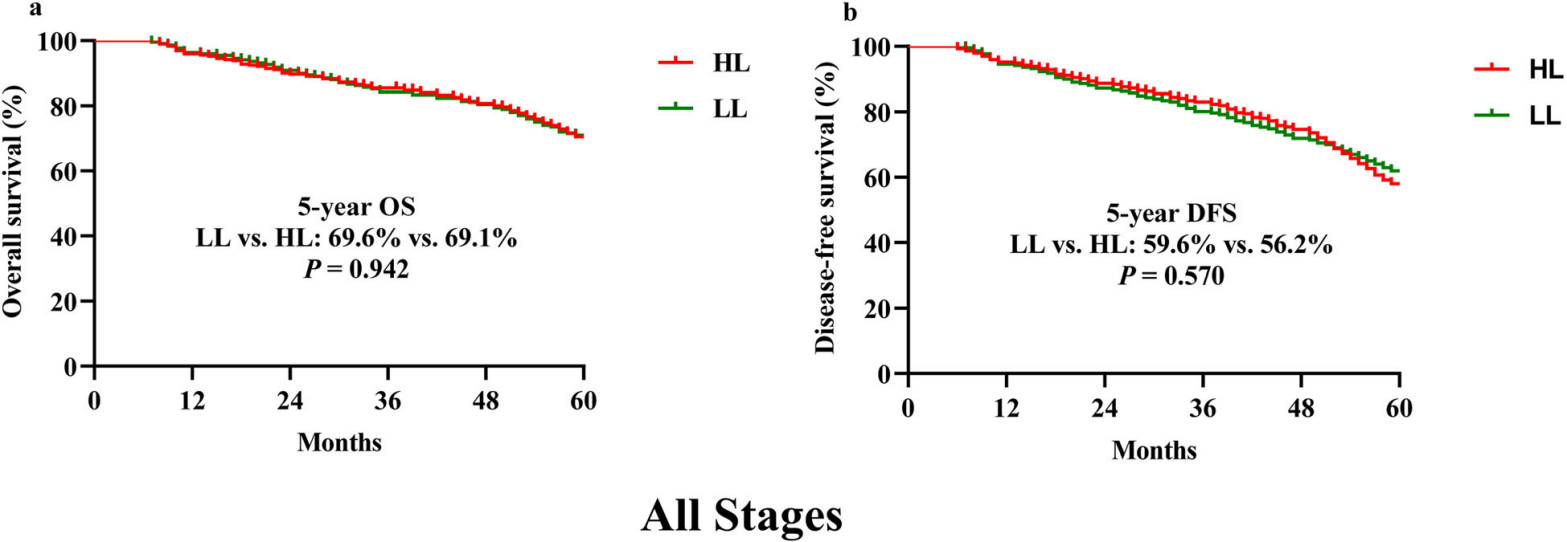
Kaplan–Meier estimates of overall survival and disease-free survival for all anterior resection cases (**a** and **b**, respectively). Abbreviations: LCA, left colic artery; HL: high ligation; LL: low ligation

**Fig. 2:**
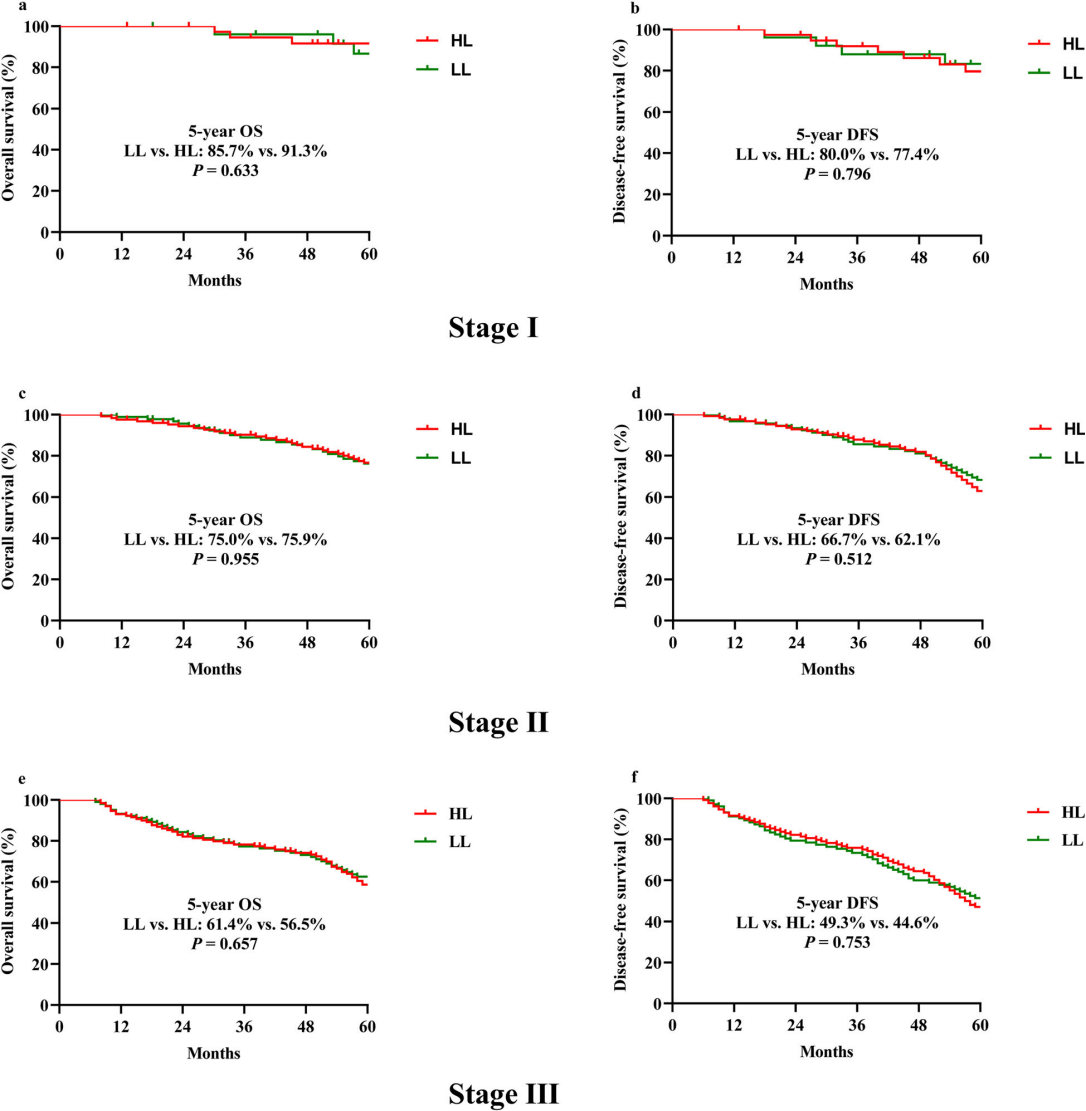
Kaplan–Meier estimates of overall survival and disease-free survival for all anterior resection cases in stage I (**a** and **b**, respectively), stage II (**c** and **d**, respectively), and stage III (**e** and **f**, respectively). Abbreviations: LCA, left colic artery; HL: high ligation; LL: low ligation

There were no significant differences in the 5-year OS and DFS (69.6% vs.69.1%, *p* = 0.942, and 59.6% vs. 56.2%, *p* = 0.570, respectively) between the LL and the HL groups, as analyzed by the Kaplan–Meier method (Fig. 1). We performed further analyses of stage-by-stage OS and DFS in stages I to III cases. There were no statistically significant differences between the LL and HL groups for these stages (Fig. 2).

## Discussion

Since Heald et al. proposed total mesorectal excision in 1982, the clinical outcomes of middle and low rectal cancer have significantly improved [18, 19]. This improvement is mainly due to reduced local recurrence [20]; however, anastomotic leakage is still an important factor affecting the short and long-term postoperative outcomes of rectal cancer, in addition to increasing the total cost of treatment [21–23]. It has been reported that the incidence of anastomotic leakage after rectal cancer surgery is between 5 and 26% [24, 25]. To date, no consensus has been reached on whether LCA preservation during rectal cancer surgery can reduce anastomosis-related complications and improve long-term prognosis [13, 26].

Excessive anastomotic tension and poor blood supply are crucial factors for anastomotic leakage [27]. High ligation of the IMA, at its aortic origin, allows a 9 cm gain of length over low ligation [28]. However, after high ligation, the proximal colon can only supply blood from the middle colic artery (a branch of the superior mesenteric artery), reducing blood perfusion of the marginal arterial arch, and disrupting the blood supply to the terminal colon [29].

Anastomotic leakage is one of the most common serious complications of rectal cancer surgery. There are many factors affecting anastomotic leakage including: the patient’s age, BMI, nutritional status, comorbidities, tumor location, neoadjuvant radiotherapy, anastomotic blood supply, and anastomotic tension [30–32]. The results of our study showed that anastomotic leakage occurred in 19 (8.6%) patients in the LL group and in 39 (13.2%) patients in the HL group. This difference was not statistically significant. Moreover, there were no statistically significant differences in anastomotic bleeding and anastomotic stenosis between the two groups. However, a meta-analysis [12], containing 17 studies with 6247 patients, showed that preserving the LCA was associated with reduced anastomotic leakage rate (odds ratio = 0.78, 95% confidence interval (CI): 0.62–0.98, *p* = 0.03). Our study may not have shown this as it was retrospective, only ISREC grade B or C anastomotic leaks could be traced back and analyzed.

Intraoperative parameters and postoperative complication rates are important indices for demonstrating the quality control of a surgical procedure. A randomized controlled trial showed that LCA preservation did not increase the duration of surgery of low anterior resection for rectal cancer [14]; however, our study revealed that the mean operating time of the LL group was significantly longer than that of the HL group. This could be due to the higher rate of splenic flexure mobilization in the LL group, prolonging the operative time for the whole group (13.1% in the LL group vs 6.1% in the HL group, *p* = 0.006). Despite the prolonged operation time in the LL group, there was no significant difference in operative bleeding between the two groups. Over 85% of the procedures were laparoscopic surgeries, with only a 2.1% conversion from laparoscopic to open surgery. After passing the laparoscopic learning curve, intraoperative bleeding does not increase significantly as the surgical area expands [33].

IMA root lymph nodes are the third station of lymphatic drainage in rectal cancer and the most important route of metastasis in progressive rectal cancer [34]. Studies have shown that IMA root lymph node metastases have a negative impact on patients’ 5-year survival and tumor recurrence rates [35]. In our study, the differences between the number of lymph nodes harvested and the number of positive lymph nodes were not statistically significant between the two groups, suggesting that the surgical approach of preserving the LCA, i.e., IMA low level ligation, does not reduce the harvesting and positivity rate of lymph nodes.

Regarding the long-term outcomes in patients, survival analysis showed that low anterior resection for rectal cancer with or without LCA preservation showed no statistically significant difference in the 5-year OS and DFS (LL group vs. HL group: 69.6% vs. 69.1 and 59.6% vs. 56.2%, respectively). Further analysis of stage-by-stage OS and DFS in stage I to stage III cases showed no statistically significant difference between LL and HL groups. Several previous studies have also shown that LCA preservation compared to non-preservation showed no significant differences with respect to the 5-year mortality in patients who underwent laparoscopic rectal cancer surgery, and this comparable success came with acceptable safety outcomes [10, 12, 13]. Data from the Japan Clinical Oncology Group Study showed that if the LCA was preserved, 5-year relapse-free survival (RFS) and OS were better than in the LCA non-preservation group (RFS: 83.7 and 80.5%, hazard ratio (HR) = 0.80, 95% CI: 0.51–1.26, OS: 96.3 and 91.1%, HR = 0.41, 95% CI: 0.19–0.89) [36]. Another Japanese study by Fujii [15] showed that the IMA ligation level was unrelated to anastomotic leakage and there was no significant difference in the long-term results between low and high ligation of the IMA. Recent meta-analyses support this finding [11, 12]. However, one meta-analysis, which included 3119 patients in five cohorts, pooled HR results showing a significant OS benefit of high ligation over low ligation (HR = 0.77, 95% CI: 0.66–0.89) [37].

Our study has certain limitations. First, this is a single-institution retrospective study, and of the 635 cases, 119 cases had missing data and were removed from the analysis. Second, Omission of circumferential resection margin positivity/negativity assessment is a weakness since this is a crucial factor that influences long-term oncological results [38]. Third, choice of surgical implemented procedure could vary between surgeons thus leading to bias in our results. Finally, there may be differences in the standards of neoadjuvant and adjuvant chemotherapies between western countries and China, which could result in diverse outcomes among patients from different countries.

## Conclusions

The results of this single-center retrospective study suggest that the early complication rates and long-term oncologic outcomes associated with LCA preservation in low anterior resection of rectal cancer are comparable with those associated with ligation of the artery at the origin of the IMA. Preserving LCA cannot be supported due to the absence of lower complication rates and the longer operating times. However, further multicenter randomized controlled trials are required to confirm the validity of these results in a broader context.

## Data Availability

The datasets used and/or analyzed during the current study are available from the corresponding author on reasonable request.

## Abbreviations

LCA: Left colic artery
IMA: Inferior mesenteric artery
HL: High ligation
LL: Low ligation
OS: Overall survival
DFS: Disease free survival
TNM: Tumor, node, and metastasis
BMI: Body mass index
ISREC: The International Study Group of Rectal Cancer
CI: Confidence interval
RFS: Relapse free survival
HR: Hazard ratio
